# Bias in Online Recruitment and Retention of Racial and Ethnic Minority Men Who Have Sex With Men

**DOI:** 10.2196/jmir.1797

**Published:** 2011-05-13

**Authors:** Patrick S Sullivan, Christine M Khosropour, Nicole Luisi, Matthew Amsden, Tom Coggia, Gina M Wingood, Ralph J DiClemente

**Affiliations:** ^3^Rollins School of Public HealthDepartment of Behavioral Sciences and Health EducationEmory UniversityAtlanta, GAUnited States; ^2^CyclogramWest Hollywood, CAUnited States; ^1^Rollins School of Public HealthDepartment of EpidemiologyEmory UniversityAtlanta, GAUnited States

**Keywords:** HIV infections/prevention and control, Internet, homosexuality male, research methodology, behavioral research

## Abstract

**Background:**

The Internet has become an increasingly popular venue for men who have sex with men (MSM) to meet potential sex partners. Given this rapid increase in online sex-seeking among MSM, Internet-based interventions represent an important HIV (human immunodeficiency virus) prevention strategy. Unfortunately, black and Hispanic MSM, who are disproportionately impacted by the HIV epidemic in the United States, have been underrepresented in online research studies.

**Objective:**

Our objective was to examine and quantify factors associated with underrecruitment and underretention of MSM of color in an online HIV behavioral risk research study of MSM recruited from an online social networking site.

**Methods:**

Internet-using MSM were recruited through banner advertisements on MySpace.com targeted at men who reported in their MySpace profile their age as at least 18 and their sexual orientation as gay, bisexual, or unsure. Multivariable logistic regression models were used to estimate the odds stratified by race and ethnicity of the MySpace user clicking through the banner advertisement. To characterize survey retention, Kaplan-Meier survival curves and multivariable Cox proportional hazards models identified factors associated with survey dropout.

**Results:**

Over 30,000 MySpace users clicked on the study banner advertisements (click-through rate of 0.37%, or 30,599 clicks from 8,257,271 impressions). Black (0.36% or 6474 clicks from 1,785,088 impressions) and Hispanic (0.35% or 8873 clicks from 2,510,434 impressions) MySpace users had a lower click-through rate compared with white (0.48% or 6995 clicks from 1,464,262 impressions) MySpace users. However, black men had increased odds of click-through for advertisements displaying a black model versus a white model (adjusted odds ratio [OR] = 1.83, 95% confidence interval [CI] 1.72 - 1.95), and Hispanic participants had increased odds of click-through when shown an advertisement displaying an Asian model versus a white model (adjusted OR = 1.70, 95% CI 1.62 - 1.79). Of the 9005 men who consented to participate, 6258 (69%) completed the entire survey. Among participants reporting only male sex partners, black non-Hispanic and Hispanic participants were significantly more likely to drop out of the survey relative to white non-Hispanic participants (hazard ratio [HR] = 1.6, 95% CI 1.4 - 1.8 and HR = 1.3, 95% CI 1.1 - 1.4, respectively). Men with a college-level of education were more likely to complete the survey than those with a high-school level of education (HR = 0.8, 95% CI 0.7 - 0.9), while men who self-identified as heterosexual were more likely to drop out of the survey compared with men who self-identified as gay (HR = 2.1, 95% CI 1.1 - 3.7).

**Conclusions:**

This analysis identified several factors associated with recruitment and retention of MSM in an online survey. Differential click-through rates and increased survey dropout by MSM of color indicate that methods to recruit and retain black and Hispanic MSM in Internet-based research studies are paramount. Although targeting banner advertisements to MSM of color by changing the racial/ethnic composition of the advertisements may increase click-through, decreasing attrition of these study participants once they are engaged in the survey remains a challenge.

## Introduction

Men who have sex with men (MSM) are the most heavily impacted risk group for HIV in the United States [1]. Indeed, MSM is the only US risk group in which HIV incidence has been increasing since 2000 [2]. Similar to the United States, there is an international resurgence in HIV infections among MSM in industrialized countries in North America, Europe, and Australia [3]. Among MSM in the United States, men of color—especially younger men of color—are experiencing the most dramatic increases in HIV surveillance case reports [4,5]. At the same time, the number of HIV prevention interventions tested with MSM is low relative to the proportion of the US HIV epidemic represented by this vulnerable population [6].

The role of the Internet in the MSM epidemic—as a facilitator of transmission or as an opportunity for HIV prevention—is complex and unclear. In the results of most studies reported to date, men who meet sex partners on the Internet are also men who are more likely to engage in high risk sex [7-10] although the causality of this relationship is not clear. Regardless of causality, the Internet seems to hold promise as a way to reach men with significant sexual risk behaviors. We also know that there have been some early suggestions that Internet-based HIV prevention interventions hold promise as a strategy for accessing and increasing protective behaviors among MSM [11].

While the Internet represents a promising intervention strategy, significant gaps remain in our ability to optimally leverage the Internet to evaluate and implement HIV prevention interventions for MSM. One of the most significant barriers is that MSM of color, who bear the greatest risk for HIV infection in the United States, are systematically underrepresented in nearly all HIV prevention Internet studies to date (Table 1). Black men have been historically underenrolled by 29% to 84%, and Hispanic men underenrolled by 6% to 89%. Setting quotas for enrollment by race/ethnicity is one way to address the problem but raises additional concerns about bias among minority men who actually enroll (and how they are different from the unsurveyed men who do not enroll). Thus, underenrollment of MSM is a problem for Internet-based HIV prevention research and is ideally addressed by finding ways to obtain comparable rates of enrollment and retention for minority men.

**Table 1:** Selected Internet-based HIV prevention studies of men who have sex with men depicting population prevalence from recruitment location, enrolled study population prevalence, and corresponding prevalence ratio of black and Hispanic men

		Black Men			Hispanic Men		
Internet Study	Location	Population Prevalence (%)	Enrolled Prevalence (%)	Prevalence Ratio	Population Prevalence (%)	Enrolled Prevalence (%)	Prevalence Ratio
Grosskopf et al, 2010 [12]	New York City	25.1	17.9	0.71	27.4	13.5	0.49
Chiasson et al, 2009 [11]	United States	12.4	6.3	0.51	15.1	14.2	0.94
Rosser et al, 2009^a^ [13]	United States	12.4	16.4	1.3	15.1	25.1	1.7
Berg et al, 2007 [14]	United States	12.4	2.5	0.20	15.1	1.7	0.11
Mackellar et al, 2007^b^ [15]	6 US cities	25.3	8.6	0.34	30.2	18.8	0.62
Chiasson et al, 2007 [16]	United States and Canada	11.3	4.6	0.41	15.1	7.1	0.57
Bull et al, 2004^c^ [17]	United States	12.4	6.6	0.53	15.1	10.9	0.72
Hirshfield et al, 2004 [18]	United States	12.4	2.0	0.16	15.1	5.5	0.36

^a^Recruitment was capped at 750 participants in each racial/ethnic group to ensure a diverse sample.

^b^The Web-Based HIV Behavioral Surveillance (WHBS) Study Group

^c^Recruitment strategies included print ads and flyers.

Our study addressed two aspects of this problem: (1) challenges in recruiting MSM of color (ie, the extent to which MSM of color click through banner advertisements to enroll in research studies) and (2) challenges in retaining MSM of color (ie, the extent to which MSM of color complete an online survey once they begin taking it). Our goals were to quantify the extent of underrecruitment of MSM of color, to identify factors associated with recruitment, and to determine the extent of underretention of MSM of color. To address these questions, we conducted an Internet survey of MSM recruited through banner advertisements shown on MySpace.com, a popular online social networking site.

## Methods

### Recruitment and Participation

Internet-using MSM were recruited from March 19, 2009, through April 16, 2009, through selective placement of banner advertisements on MySpace.com. During the recruitment period, advertisements were displayed to MySpace members based on self-reported demographic MySpace profile information. Exposures were made to males 18 years of age and over logging into MySpace whose profile indicated a residence in the United States and who reported their sexual orientation as gay, bisexual, or unsure. Participants who clicked through the banner advertisements were taken to an eligibility screener for an Internet-based survey.

**Figure 1:**
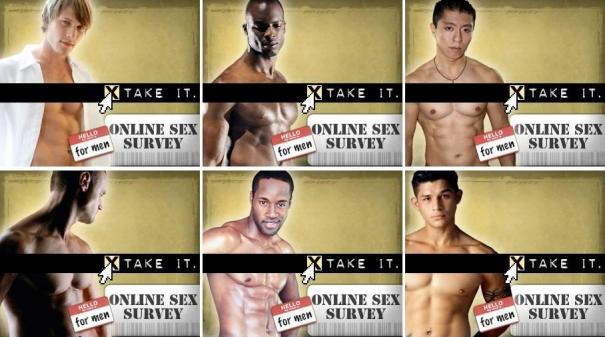
Shown are six banner advertisements displaying white (left), black (middle), and Asian (right) models used to recruit potential participants from MySpace.com for an online HIV behavioral risk study in the United States in 2009

A total of six banner advertisements were used, all with similar text and graphical design (Figure 1). Of the six, two of the advertisements presented a white male model, two presented a black male model, and two presented an Asian male model. Asian male models were used as controls since our hypotheses centered primarily on black/white differences. Data on the number of advertisement exposures and “click-throughs” were collected for each combination of model race, respondent race/ethnicity, respondent sexual orientation (gay, bisexual, unsure), and level of education (less than high school, high school, some college, college graduate and higher). Participant data on race/ethnicity, sexual orientation, and level of education for the “click-through” analysis were obtained from participants’ MySpace.com profiles. Therefore, these categories, which were preset options on MySpace.com, are not identical to those that are presented in the analysis of survey completion, where we used the data from participants’ survey responses (and which contained different category options for sexual identity and education level).

Participants referred to the survey site after clicking through the banner advertisement were first screened for eligibility. Participants were eligible for the survey if they were male, 18 years of age and over, and reported at least one male sex partner in the last 12 months. Eligible participants were provided an online informed consent document, and consenting participants were passed into an online survey. In the survey, participants were asked for relevant demographic information as well as questions about the use of the Internet to meet sex partners, recent sexual risk behaviors, use of technology, HIV testing history, and interest in specific, new HIV prevention interventions. Participants did not receive an incentive to participate in the baseline survey. The study was reviewed and approved by the institutional review board (IRB) of Emory University.

### Analysis

#### Characteristics Associated With Click-Through of Advertisements

Internet advertisement exposures and click-throughs were totaled and stratified by race/ethnicity of the MySpace user. Within each stratum of race/ethnicity, data were summarized by race of the model displayed in the advertisement, education, and sexual orientation of the respondent as reported in the MySpace profiles. We utilized multivariable logistic regression to model factors associated with clicking through the banner advertisement, stratified by race/ethnicity of the MySpace user. With click-through as the outcome and race of the model, education, and sexual identity as the independent factors of interest, we calculated adjusted odds ratios (ORs) with 95% confidence intervals (CIs) for each racial/ethnic group. 

#### Characteristics Associated With Survival in the Survey

To determine whether there was any bias in completion of the survey, we used survival analysis to model factors associated with survey dropout. We evaluated survey dropout by race, age group, sexual orientation, education, geographic region, and gender of sexual partners in the last 12 months (male only or male and female) as reported in the online survey. Participants were assigned a *complete* or *incomplete* status for each page of the survey. A *complete* status was assigned if the participant answered at least 50% of the questions on the survey page. Failure (dropout from the survey) was defined as at least two consecutive incomplete pages. Kaplan–Meier survival methods and log-rank tests were used to evaluate survey dropout by individual demographic factors. For multivariable analysis, Cox proportional hazards regression was used to identify variables associated with survey dropout. Models were constructed separately for men who had only male partners and those who had both male and female partners; the reason for this was that men with male and female partners were asked additional questions about their female partners, which resulted in a longer survey. Thus, it was not possible to equitably assess dropout in one model that included both groups. The multivariable model findings are reported as hazard ratios (HR) with 95% CIs. All data analysis was completed using SAS version 9.2 (SAS Institute, Cary, North Carolina, USA).

## Results

**Figure 2:**
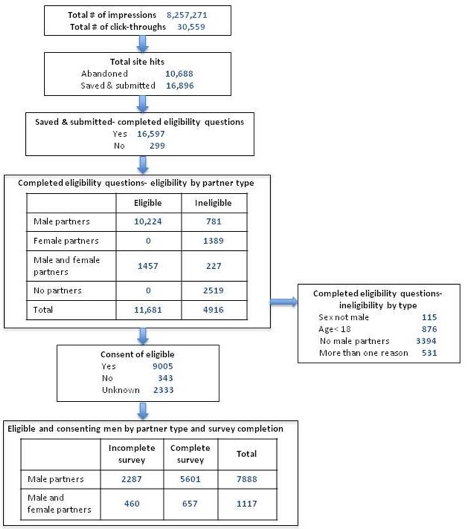
Flow chart of participant recruitment, eligibility, and enrollment in an online HIV behavioral risk study conducted in the United States in 2009

The recruitment and enrollment of study participants is described in Figure 2. A total of 8,257,271 MySpace advertising impressions over a 29-day period resulted in 30,559 (0.37%) click-throughs to our eligibility screener. Of those potential participants, 35% (10,688 of 30,559) abandoned the page without attempting the survey. Of the 16,896 respondents who saved or submitted their survey responses, 98% (16,597 of 16,896) completed the three sex, age, and partner questions used to determine eligibility. Approximately 30% (4916 of 16,597) of respondents who completed the screening questions were ineligible. Ineligibility was most often due to reporting no male sex partners in the past year (3394 of 4916 potential participants or 69%), followed by reporting an age of less than18 years (876 of 4916 potential participants or 18%). Thus, a total of 11,681 men were eligible for participation and were referred to informed consent. Another 20% (2333 of 11,681) of participants discontinued the survey at this point, not completing the informed consent process. Of those who completed the consent process, the vast majority, 96% (9005 of 9348), consented to participate.

Of the 9005 consenting participants, 39% (3473 of 9005) were white non-Hispanic, 14% (1293 or 9005) were black non-Hispanic, and 31% (2809 of 9005) were Hispanic. The median age of participants was 21, with 68% (6157 of 9005) of participants aged 18 to 24. Most reported having only male sex partners, and 12% (1117 of 9005) reported both male and female partners. In all, 69% (6176 of 8409 reporting) of the men self-identified as homosexual or gay, while 23% (2077 of 8409 reporting) self-identified as bisexual, and less than 1% (60 of 8409 reporting), as heterosexual or straight. Over half of participants (4815 of 8357 reporting) attended at least some college, and about a third (2914 of 8357) completed high school or a general equivalency diploma (GED). Over 60% of participants (4843 of 8063 reporting) had unprotected anal intercourse (UAI) with a male sex partner in the past 12 months, while over a quarter of participants (1775 of 6623 reporting) indicated they had never been tested for HIV.

### Characteristics Associated With Click-Through of Advertisements

The overall click-through rate for the banner advertisements was 0.37% (30,599 clicks from 8,257,271 impressions); this varied by race, education, sexual identity, and race of model. In general, black, Hispanic, and men of other races were less likely to click through on the banner advertisements than were white men, with click-through rates of 0.36% (6474 clicks from 1,785,088 impressions) for black men, 0.35% (8873 clicks from 2,510,434 impressions) for Hispanic men, and 0.33% (8271 clicks from 2,497,487 impressions) for men of other races versus 0.48% (6995 clicks from 1,464,262 impressions) for white men. Men with more than a high school level of education were more likely to click through, as were men who self-identified as gay or bisexual. Click-through rates were higher when the models displayed were black (0.33% or 9006 clicks from 2,751,415 impressions) or Asian (0.48% or 13,241 clicks from 2,753,684 impressions) versus white (0.30% or 8312 clicks from 2,752,172 impressions) (data not shown).

Because our primary research question was focused on how to optimize banner advertisement to achieve comparable click-through rates for racial/ethnic minority men, we conducted multivariable analyses stratified by race/ethnicity (Table 2). Among all nonwhite racial/ethnic groups analyzed, men with greater than a high school education had higher odds of click-through compared with those with less education. Black, white, and Hispanic men that self-identified as gay or bisexual also had higher odds of click-through. Hispanic men had higher odds of click-through when exposed to Asian models; black men had higher odds of click-through when exposed to black models. White men had higher odds of click-through when shown an Asian model, but lower odds of click-though when shown a black model. 

**Table 2:** Odds of clicking on study banner advertisements by MySpace.com users controlling for self-reported education, sexual identity, and race of model in advertisements and stratified by race of the MySpace.com user in the United States in 2009

		White Men	Black Men	Hispanic Men	Other Men
Characteristic		Adjusted OR (95% CI)	Adjusted OR (95% CI)	Adjusted OR (95% CI)	Adjusted OR (95% CI)
**Education**					
	< High School (referent)				
	> High School	0.99 (0.95 - 1.04)	*1.20 (1.14 - 1.26)*^a^	*1.05 (1.01 - 1.10)*	*1.10 (1.04 - 1.16)*
**Identity**					
	Unsure (referent)				
	Gay	*2.10 (1.98 - 2.24)*	*1.62 (1.53 - 1.71)*	*1.45 (1.38 - 1.52)*	*3.07 (2.88 - 3.28)*
	Bisexual	*1.63 (1.53 - 1.74)*	*1.78 (1.67 - 1.89)*	*1.58 (1.49 - 1.67)*	*2.83 (2.63 - 3.04)*
**Race of model**					
	White (referent)				
	Black	*0.74 (0.70 - 0.79)*	*1.83 (1.72 - 1.95)*	1.05 (0.99 - 1.11)	0.95 (0.89 - 1.00)
	Asian	*1.56 (1.47 - 1.64)*	*1.46 (1.37 - 1.56)*	*1.70 (1.62 - 1.79)*	*1.61 (1.52 - 1.69)*

^a^Results presented in italics denote significance at *P* < .05.

### Characteristics Associated With Survival in the Survey

**Figure 3:**
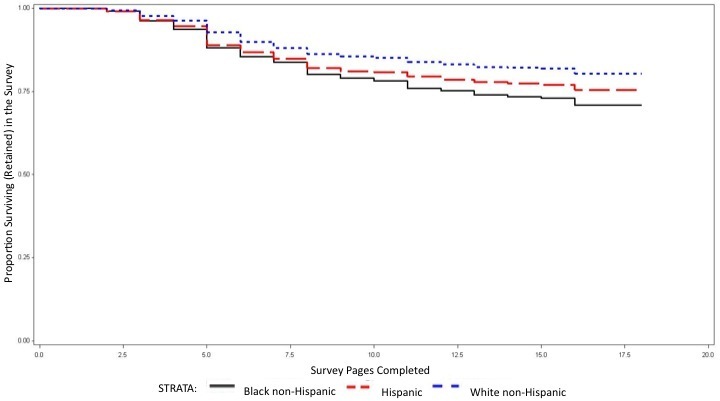
Retention in an online behavioral risk survey among participants reporting only male partners in the past 12 months, by race/ethnicity of the participants in the United States in 2009

**Figure 4:**
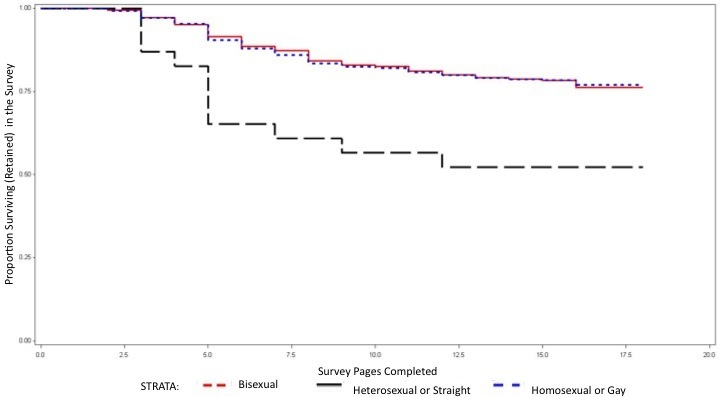
Retention in an online behavioral risk survey among participants reporting only male partners in the past 12 months by self-identified sexual orientation of the participant (United States, 2009)

Of the 9005 participants, 69% (6258 of 9005) completed the entire survey, and 31% (2747 of 9005) dropped out of the survey after starting. Demographic characteristic of participants by survey completion status are provided in Table 3, and hazards of dropping out of the survey from the Cox proportional hazards model are described in Table 4. Figures 3 and 4 display the Kaplan-Meier curves for survival in the survey by participant race/ethnicity and sexual orientation, respectively, among participants reporting having only male partners in the past 12 months. Among white study participants, 77% (2670 of 3473) completed the entire survey compared with 66% (849 of 1293) of black participants and 71% (1987 of 2809) of Hispanic participants. Among those with only male sex partners in the last year, black men had about a 60% higher hazard of dropping out than white men, and Hispanic men had an increased hazard of about 30%. Among those with both male and female partners in the last year, both black men and Hispanic men had an increased hazard of dropout (30% and 50% increased hazard, respectively) compared with white men. Only 58% (35 of 60) of men who self-identified as heterosexual completed the survey compared with 75% (4653 of 6176) of men who self -identified as gay, resulting in heterosexually identified men having over twice the hazard of dropping out of the survey compared with men identifying as homosexual. Participants who reported less than a high school level of education had an increased hazard of dropout compared with those with greater than a high school level of education. There was no significant difference in hazard of dropout related to age or geographical region.

**Table 3:** Demographic characteristics of survey participants enrolled in an online HIV behavioral risk study by survey completion status (n = 9005) in the United States in 2009

		Completed Each Page in Survey (n = 6258)		Did Not Complete Each Page in Survey (n = 2747)	
	Characteristics of Participants^a^	n	%	n	%
**Race**					
	White^b^	2670	77	803	23
	Black^b^	849	66	444	34
	Hispanic	1987	71	822	29
	Asian/Pacific Islander^b^	138	66	70	34
	Native American/Alaska Native^b^	123	67	61	33
	Multiracial^b^	332	73	120	27
	Other^b^	100	75	33	25
**Age (years)**					
	18-24	4181	68	1976	32
	25-29	1014	70	432	30
	30-34	431	75	142	25
	35-45	477	77	146	23
	> 45	155	75	51	25
**Sexual identity**					
	Bisexual	1445	70	632	30
	Homosexual or gay	4653	75	1523	25
	Heterosexual or straight	35	58	25	42
	Other^c^	70	73	26	27
**Education**					
	College/postgraduate	969	75	315	25
	Some college/associate degree	2694	76	837	24
	High school or GED	2082	71	832	29
	Less than high school	444	71	184	29
**Sexual Partners, past 12 months**					
	One or more men	5601	71	2287	29
	Both men and women	657	59	460	41
**UAI with a male sex partner, past 12 months**					
	Yes	4064	84	779	16
	No	2189	68	1031	32

**Ever been tested for HIV**					
	Yes	4501	95	258	5
	No	1674	94	101	6
**Urban versus rural**^d^					
	Rural	2376	75	805	25
	Urban	3608	72	1381	28

^a^ Totals for most variables do not equal the total number of participants due to missing data.

^b^ non-Hispanic

^c^ Participants could write in a text response for “Orientation”; the most frequent responses were “queer,” “curious,” and “questioning.”

^d^ The categorization of rural versus urban was based on population density (per square mile) of the respondents’ zip codes; respondents who lived in a zip code with a population density of < 1000 persons per square mile were considered to live in rural areas.

**Table 4:** Hazards of failure to complete all pages of an online HIV behavioral risk survey, by gender of sexual partners of participants in the past 12 months in the United States in 2009

		Male Partners Only	Male and Female Partners
Characteristics of Participants		Hazard Ratio (95% CI)	Hazard Ratio (95% CI)
**Race**			
	White^a^ (referent)		
	Black^a^	*1.6* (*1.4-1.8*)^b^	1.3 (1.0-1.8)
	Hispanic	*1.3* (*1.1-1.4*)	*1.5* (*1.2-1.9*)
	Other^a^	*1.3* (*1.1-1.5*)	1.0 (0.8-1.5)
**Age(years)**			
	18-24 (referent)		
	25-29	1.0 (0.9-1.1)	1.0 (0.8-1.3)
	30-34	0.9 (0.8-1.1)	0.7 (0.4-1.1)
	35-45	0.8 (0.7-1.0)	0.8 (0.5-1.2)
	> 45	0.8 (0.6-1.1)	1.0 (0.5-2.2)
**Sexual identity**			
	Homosexual or gay (referent)		
	Bisexual	1.0 (0.8-1.1)	0.8 (0.6-1.1)
	Heterosexual or straight	*2.1* (*1.1-3.7*)	0.8 (0.4-1.4)
	Other^c^	0.9 (0.5-1.5)	0.6 (0.3-1.1)
**Education**			
	College/postgraduate	*0.8* (*0.7-0.9*)	0.9 (0.7-1.3)
	Some college/associate degree	*0.8* (*0.7-0.9*)	1.0 (0.8-1.3)
	High school or GED (referent)		
	Less than high school	1.0 (0.8-1.2)	1.3 (0.9-1.9)
**Urban versus rural**^d^			
	Rural	0.9 (0.8-1.0)	1.2 (1.0-1.5)
	Urban (referent)		

^a^ non-Hispanic

^b^ Results presented in italics denote significance at *P* < .05.

^c^ Participants could write in a text response for “Orientation”; most frequent responses were “queer,”“curious,” and “questioning.”

^d^ The categorization of rural versus urban was based on population density (per square mile) of the participants’ zip codes; participants who lived in a zip code with a population density of < 1000 persons per square mile were considered to live in rural areas.

## Discussion

Our results illustrate two levels at which bias may occur in online HIV prevention research with MSM. Based on differential click-through rates, our data suggest that online surveys recruited through banner advertisements similar to ours may underrepresent black and Hispanic MSM, MSM with less education, and MSM who do not identify as gay. Once engaged in the survey, further bias is introduced by differential dropout during the survey; in this case, our data suggest that we were more likely to lose as participants men who were black, Hispanic, or of other nonwhite races, as well as men who were less educated, and men who identify as heterosexual.

Our approach furthers knowledge in this field because we used an Internet venue for advertisement in which we could collect data on each exposure to the banner advertisement (each impression) along with the race, education, and sexual identity of the person who saw the advertisement (as reported in their profile). This allowed us, for the first time to our knowledge, to calculate race-, education-, and sexual identity-specific click-through rates and model how the characteristics of Internet users who saw the banner advertisements related to their likelihood of clicking through that advertisement. The results indicate that the black men who saw our advertisements were much more likely to click through an advertisement displaying a black model than one displaying a white model, whereas the white men who saw our advertisements were much less likely to click through an advertisement with a black model than a white model. All of the racial/ethnic groups were more likely to click through on the advertisements displaying the Asian models compared with the advertisements displaying the white models; however, it is unclear why the Asian model advertisements yielded the highest click-through.

These data suggest that it may be possible to mitigate the differentially low click-through of black and Hispanic MSM by changing the racial/ethnic composition of the banner advertisements that they are shown. For example, black Internet users may be shown, as a group, banner advertisements that predominantly depict black models. It is important to emphasize that, to our understanding, an important goal is to achieve comparable click-through rates by racial or ethnic group. It will always be possible to set quotas to ensure that equal numbers of men in each racial/ethnic group are included, but the central question must be what biases attend the inclusion of those specific men. In other words, if 25% fewer black men are clicking through than white men, how are those men who are not clicking through different from the ones who are? Striving for comparable click-through rates by race/ethnicity is one way to address this concern.

Although MSM of color were also more likely to drop out of our survey prematurely, our data are less definitive in identifying solutions to this source of bias. However, we propose several possible reasons for our findings and options to reduce differential dropout. First, it is well documented that black Americans are less likely to have access to private, high speed Internet than are white Americans [19]. We speculate that, if black non-Hispanic participants were taking the current survey either without high-speed Internet access or in a public place (for example, a library), they may have discontinued participation early because of frustration with page load times or because of privacy concerns as questions became more personal. Possible solutions include optimizing our Internet interfaces for nonbroadband use or allowing an option to complete surveys via text messaging, as black and Hispanic Americans exceed white Americans on mobile phone ownership and text message use [20]. Another possible explanation for our results is that participants with less education drop out sooner because their literacy skills are lower. Possible solutions are attempting to lower the reading level of the survey and adding an option for text-to-speech service to the survey website. An additional explanation may be that the sensitive nature of the questions (ie, sexual history and HIV testing information) may have led some participants to discontinue their participation. An informal analysis of the pages from which men dropped out of the survey did not indicate that dropouts were clustered on a certain page; therefore, if the content of questions was a cause for dropout it was more likely due to the general nature of the topics and not to a specific question. However, it is important to note that bisexual- and straight-identified participants, although they had already reported having male sex partners, tended to drop out when the series of questions about male partners began. In future versions, we may choose to ask questions about male sex partners later in the survey, after questions about female sex partners.

Although not the primary focus of our study, we also note that social networking sites may be fruitful places for HIV prevention research among MSM. Most previous studies have recruited from sites that were either more explicitly gay-identified [13,16] or that were sex-seeking sites [11]. Using MySpace, we found that we were able to recruit a large number of behaviorally eligible MSM quickly. Also, the demographic and behavioral characteristics of these men suggest that they were at least as high risk and in need of prevention services as men recruited in “‘real-world” venues. The greatest expansion in the US HIV epidemic among MSM is among MSM aged 13 to 24. The median age of our consenting participants recruited through a social networking site was 21; for comparison, the average age of participants in the 2008 National HIV Behavioral Surveillance System (NHBS) was 32 [21]. Also, our participants were relatively high risk: 60% reported unprotected anal intercourse with a male sex partner in the past 12 months compared with 47% of 2003-2005 NHBS respondents [22]. Use of prevention services among our participants was low, with 28% never having been tested for HIV versus 10% of 2008 NHBS respondents who had never been tested [21]. Thus, recruiting through a social networking site geared toward younger users yielded a young, high-risk sample with low levels of prior HIV testing—an ideal population for HIV prevention research and, eventually, Internet-delivered interventions.

There are a number of limitations to our study. First, our participants are not a representative sample, and our conclusions cannot be generalized to all MSM users of MySpace, to users of other social networking sites, or to US MSM more generally. Because we only showed our banner advertisements to men who identified themselves in their MySpace profiles as gay, bisexual, or unsure, we did not display banner advertisements to men who may have had male sex partners but who identified themselves as straight. Further, we could not verify that certain self-reported characteristics of our respondents—for example, male sex, or race/ethnicity—were correct. However, we recruited men based on their MySpace profile, and therefore we would only refer men to the eligibility screener if they had identified those characteristics in their profile, independent of the research study. Our data were subject to recall bias because of our 12-month recall period and to potential social desirability bias. There is also concern about participants taking the survey multiple times. In our case, we think that this was not common for two reasons. First, participants could only enter the survey site by being referred from a link in the banner advertisement. Therefore, a participant could only participate a second time if he was shown the banner advertisement a second time—a low probability event. Also, we did not allow multiple surveys to be completed from the same Internet protocol (IP) address, which would further require that a participant change his IP address, or take the survey a second time from a different computer. 

Our study has several implications for those conducting online HIV prevention research with MSM. First, we have demonstrated that it is possible in some Internet settings to collect denominator data, characterize nonrespondents (ie, men who clicked on the advertisement but did not answer any survey questions), and model factors associated with participation. Second, our data suggest that social networking sites may offer an appropriate alternate recruitment venue to sex-seeking sites, especially for studies that seek to enroll younger, higher risk MSM. Finally, our results suggest the need for further work to attempt to reduce disparities in click-through rates for MSM of color and improve retention in surveys of MSM of color, of those with less education, and of those who are not gay-identified. These are difficult problems to address, but the epidemiology of the MSM epidemic in the United States demands that we address them, and increasing technological capacities of Web services where MSM congregate offer new opportunities to apply the highest standards of prevention research to online HIV prevention studies.

## Abbreviations

CI: confidence interval
GED: general equivalency diploma
HIV: human immunodeficiency virus
HR: hazard ratio
IP: Internet protocol
IRB: institutional review board
MSM: men who have sex with men
NHSB: National HIV Behavioral Surveillance System
OR: odds ratio
UAI: unprotected anal intercourse
